# Enemies of the Human Teeth

**Published:** 1894-04

**Authors:** A. M. Benson

**Affiliations:** La Crosse, Wis.


### Enemies of the Human Teeth.

BY A. M. BENSON, M.D., D.D.S., LA CROSSE, WIS.

Read in the Section on Dental and Oral Surgery, at the Forty-fourth Annual Meeting of the American Medical Association.

In the same mail with Dr. Talbot's letter of invitation to prepare a paper for this meeting, came a circular from a member of our profession announcing that he had discovered a new local anesthetic, making the extraction of teeth harmless and painless, the greater part of it being devoted to testimonials from doctors and dentists who had used it, and telling how they had removed from six to twenty teeth from the mouth of one person at a time, thereby very much increasing their business, the author closing with urgent advice to all dentists to avail themselves of this great boon offered to the profession at the very low price of one dollar a bottle or six for five dollars. During the past year I have received perhaps half a dozen similar advertisements, and nearly all of them signed by a D.D.S. Many of you have had no doubt a like experience.

Chagrined at this impudence and unprofessional proceeding, and believing that such practice ought not to go uncondemned and under professional protection, I have accepted the invitation, and the enemies I wish to direct your attention to are not those about which so much has been written, which it takes a scientist with a microscope to discover, and against whom the evidence is mostly circumstantial, but those conspicuous and more dangerous ones whom we have all seen with the naked eye, and many of us felt upon our naked jaw; who plume themselves with professional titles and dwell mostly in "dental parlors," though they are sometimes found in the country doctor's office; who shed innocent blood and mutilate the human body in the guise of saviors, and claim special social recognition and honor; who write articles for the journals and are often found in college faculties, where they are conspicuous for their zeal in elevating the standard of the profession; whose interesting portraits so often confront us in the advertising columns of the public press along with the rest of the medicine humbugs.

Perhaps some one murmurs: This is a slander. Let me relate what I witnessed in one of the high-grade schools:

A local dentist, not a member of the faculty, was invited to give a clinique for the benefit of the class, the operation to be the extracting of a number of teeth under an anesthetic, ether being the one administered. The victim, an ignorant servant girl, whose mouth was indeed in great need of renovation, affording the students a splendid opportunity to take a lesson in the highest achievement of the dentist, "the restoration to usefulness of diseased teeth." But what was done under the direction of the professor? The frightened girl was placed in the chair, pulled into the recumbent position, and the inhaler placed over her face, against all of which she frantically struggled, but the odds being against her, she finally succumbed. Then began a scene worthy of a place in the Spanish Inquisition, and which properly described and illustrated would form a fitting canto in Dante's "Hell." The chief operator, after removing his coat and cuffs, seized a forceps and with trembling hand thrust it into the spongy gums as near a tooth and collection of tartar as his shaky condition would permit. Successive quick jerks, accompanied by the sounds of crushing bones and low groans, followed by a swaying among the pressing crowd, trying to dodge flying pieces of haggled humanity, continued for some time, when, owing to exhaustion of the operator or the overflowing of blood and ingesta which threatened to strangle the patient, active operations were for a moment suspended; but there still remaining some roots deep in the gaping wounds of her jaws, others present knowing themselves experts volunteered to kindly assist the tired scientist and cover themselves with blood and glory. The former was easily accomplished, but the opportunities for glory seemed wanting, and after bringing away a few chunks of gum and process, they retired, declaring those were the most obstinate roots they had ever encountered. The patient having again collapsed, a sympathetic looker-on, realizing if this poor creature should any longer serve the ends of science she must die, suggested that the clinique cease for the present, and the remaining roots be removed at a future time, when the gums were not so swollen. The hour, too, now having passed, the class, evidently satisfied that their alma mater had provided them a fine clinique, hurried away to the amphitheater to listen to a lecture on remote disorders in the body caused by carious teeth, leaving the patient to the care of the janitors, who, considering her from the human rather than scientific standpoint, kindly cared for her until she was somewhat restored, and some hours later, though still dazed and bleeding, conducted her out from this woeful place.

I have never been able to conceive of any excuse for such an outrage within a dental college. As evidence that such teaching bears abundant fruit in practice, I quote, verbatim, articles from a popular dental journal, the first headed: "A Large Tooth."

"About a month ago a lady from our town introduced herself to me with the intention of having some teeth extracted to make room for an artificial set. I removed eight of them with great difficulty, though I had administered first a soothing preparation invented by me for this purpose. A few days ago the lady returned to have the remaining teeth removed. In examining the mouth, I found a perfectly sound upper cuspid on the right side, and I advised her to let it remain. The lady, however, insisted on having it removed, in doing which I succeeded, after two unsuccessful attempts, with safety forceps. The tooth is one and one-eighth inches long, and shows a circumference of one inch. The lady weighs at the present time 110 pounds. Has any one extracted a larger tooth? We should like to compare notes."

In the next issue appeared the following answers:

"About five years ago I was practicing in the town of ——. One day a cadaverous-looking man came into my office to have several teeth extracted. After removing two or three, I tackled the right upper cuspid, and after some little difficulty it gave way. I thought I had pulled the whole jaw off, for a large piece of process and the first bicuspid came with it. The bicuspid is one and seven-sixteenth inches long and one and one-sixteenth inches in circumference."

"Last June I extracted seven superior teeth for a lady. I found four very difficult to remove, viz: two canines and two first bicuspids. One canine is one and five-sixteenth inches long and fifteen-sixteenths of an inch in circumference. The bicuspids were bayonet-shaped. One is one and one-sixteenth inches long and one inch in circumference. The lady weighs about one hundred and twenty pounds."

"About three weeks ago I extracted six teeth for a lady, among which were two upper cuspids, both largely decayed. The larger of the two measured one inch and five-sixteenths in length and almost one inch in circumference. The smaller is one and a quarter inches long by fifteen-sixteenths of an inch in circumference. I preserved these monsters and have them in my possession."

I avoid names, as my desire is not to point out individual shortcomings, but to direct attention to a too common practice. I believe the venerable Dr. Holmes is credited with saying, that if all the drugs used as medicine had been sunk in the bottom of the sea, it would have been better for humanity in general. I think the same might be said, if not of the entire dental profession up to date, at least of all the instruments invented for the extraction of teeth.

I venture the statement that at the present time, though in the vanguard of the profession are many noble men who can truly be called dental doctors, a large number of so-called dentists are truly "enemies of the human teeth," destroying more than they save.

I knew a dentist, now deceased, who during the twenty-five years he was in practice extracted, it is safe to say, enough teeth to fill an old-fashioned farm cart. Imagine for a moment, what this represents in blood, tears and trepidation, to say nothing of the leering deformity, crippled condition, and many deaths directly due to this malpractice. The best men in the profession are agreed that 99 out of 100 of all the teeth and roots presented for treatment can be saved and made comparatively useful, and with less pain to the patient.

It has also been demonstrated by implantation—though I

hope this operation will not become popular—that teeth long out of the mouth and strangers to the new organism can be accepted and become firmly held in the jaw; then how much better must it be to retain roots which have the advantage of natural position and long-established attachment. This being true, what shall we say of those who extract sound teeth to make room for an artificial set, or because some people ignorantly desire it?

As mentioned in the beginning of my paper, this has all been said before, and the majority of dentists have a vague idea that it is wrong. Then why is it? I think the chief reason may be given in one word, "Business."

O, Business! what crimes are committed in thy name!

The public has so long been educated by the quack in all the departments of life, that it is easy for men without moral character, but possessed of some cunning, to get money by preying upon them. Every day thousands of people freely give hard-earned money to have teeth extracted which should be saved, and thousands of dentists daily serve them, conscious of the wrong, simply because they want the money. Alas, that cash should be so dear and flesh and blood so cheap!

A dealer in dental materials informed me that one manufactory of artificial teeth sent out 5,000,000 sets each year, and there are a number of such enterprises in our country whose prosperity depends mostly upon the efforts of those dentists who extract teeth to make room for their products. Unfortunately this weakness for money does not belong to the dental specialty alone. If from all the learned professions were taken those who practice for revenue only, the number in each would probably be very much diminished.

Theoretically, this is an age of high ideals, but practically we have something yet to reach; and though I have called attention to evils common in our profession, and am convinced that for many of us it were well to examine ourselves if we are worthy the title we bear, yet I believe that our specialty has made greater progress than any other department of medical science, having accomplished the successful treatment of nearly all the diseases of the mouth, and making the preservation of teeth in the hands of the intelligent and conscientious dentist a certainty. In my opinion what is needed is not so much higher knowledge, but a better use of that already possessed.

While at college I was often edified with encouraging lectures by members of the faculty who never failed to inform us that there was plenty of room at the top of the ladder, seldom mentioning that there was anything needful or honorable to do at the bottom.

This may be well, but I have discovered in practice that if the glory of the dentist be the salvation of the natural teeth, for the dentist true to the noblest impulses of his nature who values the approval of his own better judgment above all things, and will succeed by honorable means or else retire, there is an extensive field for labor at the bottom of our professional ladder. Probably less than one-fourth of our population take proper care of their teeth.

I have thought that if, as in the clerical profession, we had different degrees or orders in our specialty, so that the ambitious youth who now seeks admission to the college, but owing to its high requirements fails to enter; for such there might be a sort of preparatory department where, if bringing evidence of having learned their catechism (especially our duty to our neighbor) they might be admitted and instructed in that branch of our work which does not necessarily require an extensive knowledge in science and literature, but rather mechanical skill, which having learned, they might be ordained and licensed like *Fourier's Sacred Band*, to do such duties as are distasteful and likely to be neglected by the D.D.S.—missionaries, as it were, to a benighted public, who now under the flickering gasoline light sit and allow these devils who go up and down our country, seeking whom they may devour, to scatter their teeth about the public market-places while the band plays " Annie Rooney "—saving them from this quack and his brethren, the vitalized-air and local-anesthetic fiends, by teaching the use of the tooth-brush and the saving power of amalgam filling, and after thus having served humanity for some time, and honestly earned some money by only asking the same fee for saving teeth as is now willingly paid for destroying them, might take a further course at college.

Three hundred years before Christ, Erasistratus, a dentist of ancient Greece, is said to have deposited in the temple of the Delphine Apollo, a leaden tooth-forceps, to impress upon all beholders that only those teeth should be extracted which could be removed with such an instrument. Might not, in our day, such an emblem be profitably suspended in every church and public school-house, or at least in every medical college and dental office?

But let me bring this paper to an end, confessing that its only redeeming feature is the object for which it was written, and if it shall be the means of saving a single tooth which otherwise would have been destroyed, I shall believe it was not written in vain.

Dr. Edgar Palmer said that the indiscriminate use of these secret compounds for local anesthesia should be absolutely and sternly discountenanced. He had never used any such. He had very carefully and rarely used a formula which had been recommended to him by one he could trust. He knew of cases where septicemia had been caused by hypodermic injections of such a preparation by one who was exhibiting it for advertising purposes, causing one death and severe trouble to several.

Dr. Taft said that every dentist should stamp his disapproval on everything which is brought out as a nostrum, and never lose an opportunity to warn people of the dangers of the traveling quack who goes from town to town, and either from the gaudy chariot or in the "dental parlor" extracts teeth without pain. The danger of operating on one patient right after another, using the same syringe and the same forceps is so serious that if it were understood their occupation would be gone. Besides the danger of infection, is the necessity of having the nostrum strong enough to be effective in every case; this means that for those who would be easily influenced it is dangerously strong. People should be intelligent enough to know that they should not sacrifice teeth except when they can not be saved, and that one whose business is only to extract is not working for the good of the patients, but of his own pocket. The profession should set its face as a flint against such practices, and treat such operators as the Irishman treated the crowd in the fight, "whenever you see a head, hit it," and crush it if possible.

Dr. S. Saxe spoke of a man who came to his town, and, with a grand flourish of advertisements, announced himself as a famous dentist, and agreed to extract all teeth painlessly, and to refund the money unless he did so. The quack referred to, who really was a graduate and sported the D.D.S. after his name, pocketed over $300 a week, while the conscientious dentists in the town were helpless to hinder the harm he did. The only way the dentists can stop such disastrous proceedings is to educate the people as to the value of the natural teeth, and the means necessary to preserve their usefulness.

Dr. Brown said he knew of one of these painless-extracting fiends, who, having guaranteed to extract with absolutely no pain, had been sued for damages by several sufferers upon whom the anesthetic had not had the desired effect, and he had had to pay the damages and seek new fields for practice.

Dr. Edgar Palmer said, on account of his position in the State Society, he always heard of these cases as soon as they advertised, and that he had taken pains to discover that this man was a graduate of the University of Pennsylvania. Upon learning this, he had written to Dr. Darby, sending him a copy of the advertisement. Dr. Darby had replied that he had communicated with the fellow, and would let him know what he said when he received his reply.

Dr. Taft said we can not hold the institution which graduates a man responsible for his future acts, and after a man has graduated the institution can exercise very little control over him. In some States the law will take hold, but in most States we can do nothing.

Dr. Benson thought that in cities the boards of health should prevent such practices. The ignorant public should be protected from the loss of their teeth, which loss will surely result in danger or injury to their future health.

Dr. V. A. Latham thought that the diploma should contain an agreement that advertising or other grossly unprofessional conduct would work a revocation of the diploma. This was the case in Great Britain, where a dentist who had received a diploma from any of the institutions which issued them, and who should be convicted of unprofessional conduct, would have his diploma annulled, his name stricken from the register, and would be fined or imprisoned should he describe himself as dentist, or use the initials of his degree after his name.

